# Design and application of an electrochemical cell for *operando* X-ray diffraction and absorption studies for electrocatalysts

**DOI:** 10.1107/S1600577525005612

**Published:** 2025-08-15

**Authors:** Jiajun Chen, Zhenzhong Li, Huiyan Zeng, Liting Deng, Long Gu, Chao Wang, Chunzhen Yang, Dongbai Sun

**Affiliations:** a School of Materials Sun Yat-Sen University Shenzhen518107 People’s Republic of China; b School of Materials Science and Engineering and Southern Marine Science and Engineering Guangdong Laboratory (Zhuhai) Sun Yat-Sen University Guangzhou510006 People’s Republic of China; Utrecht University, The Netherlands

**Keywords:** *operando* cells, electrocatalysts, *operando* synchrotron X-ray techniques, X-ray diffraction (XRD), X-ray absorption fine structure (XAFS)

## Abstract

The development of advanced catalysts for various electrochemical reactions necessitates precise characterization of their structure and dynamic evolution. This study introduces an innovative electrochemical cell tailored for *operando*X-ray characterizations, including X-ray diffraction (XRD) and X-ray absorption fine structure (XAFS). The cell features an adjustable aqueous electrolyte window to reduce X-ray signal absorption and an integrated flow system for efficient removal of gas products. This design enables simultaneous XRD and XAFS measurements in both fluorescence and transmission modes. Using LiCoO_2_ as a model oxygen evolution reaction catalyst, *operando* measurements reveal structural transformations during the reaction. This device will aid in the exploration of catalyst mechanisms and the development of high-performance catalysts.

## Abstract 2

An innovative electrochemical cell is introduced, optimized for *in situ*X-ray diffraction and X-ray absorption spectroscopy studies of electrocatalysts, featuring an adjustable aqueous electrolyte window to minimize X-ray absorption and a flow system for efficient gas product removal during *operando* testing.

## 1. Introduction

Various electrocatalytic processes, such as the oxygen evolution reaction (OER) (Hu *et al.*, 2023 ▸; Xu *et al.*, 2023 ▸; Yang *et al.*, 2020 ▸), hydrogen evolution reaction (HER) (Liu *et al.*, 2023 ▸; Xiong *et al.*, 2023 ▸), carbon dioxide reduction reaction (CO_2_RR) (Lai *et al.*, 2022 ▸; Saha *et al.*, 2022 ▸; Huang *et al.*, 2024 ▸), nitro­gen reduction reaction (NRR) (Mahmood *et al.*, 2024 ▸; Sun *et al.*, 2023 ▸), urea oxidation reaction (UOR) (Zemtsova *et al.*, 2023 ▸) *etc.*, play important roles in many energy-related technologies (Elouarzaki *et al.*, 2012 ▸). Catalysts undergo complex structural changes during reactions, including active site reconstruction, metal particle aggregation, and inter­actions between the support and active components. These changes significantly impact the catalyst’s activity, selectivity and stability. As suggested by the ‘4R–4M’ strategy (Sun *et al.*, 2024 ▸), researchers need to employ *in situ* characterization techniques to study these materials across multiple scales to obtain precise insights into their mechanical, structural and compositional properties. Understanding the dynamic structural evolution of catalysts during complex electrochemical/chemical processes is essential for elucidating their mechanisms and developing advanced materials (Lei *et al.*, 2022 ▸; Burke *et al.*, 2015 ▸; Fabbri *et al.*, 2017 ▸).

*In situ*X-ray absorption fine structure (XAFS) is a powerful technique for analyzing electrocatalysts, since it enables selective analysis of the local structure and electronic state of an X-ray absorbing atom (De Luna *et al.*, 2018 ▸; Qi *et al.*, 2023 ▸; Pearce *et al.*, 2019 ▸; Piovano *et al.*, 2011 ▸; Sun *et al.*, 2022 ▸; Zhao *et al.*, 2022 ▸). In addition, the synergy of X-ray diffraction (XRD) and XAFS measurements offers a powerful analytical approach for elucidating structural order on both macroscopic and atomic scales (Timoshenko & Roldan Cueyna, 2021 ▸; Lin *et al.*, 2017 ▸). For instance, Timoshenko *et al.* (2022 ▸) employed time-resolved XAFS and XRD with subsecond resolution to examine the dynamic evolution of Cu_2_O nanocubes under pulsing conditions. During pulsed CO_2_RR with a pulse duration and cycle time of 10 s and an applied potential of 0.6 V, both *in situ* X-ray absorption spectroscopy (XAS) and XRD confirmed the periodic and reversible changes in the catalyst’s structure and the composition of Cu species. These studies highlight the significance of *operando* XRD and XAS techniques in unraveling the complex interplay between catalyst structure and reactivity (Fabbri *et al.*, 2017 ▸).

To advance *operando*X-ray characterization techniques, several specialized electrochemical cells have been developed for various catalyst studies. For example, Lopez-Astacio and co-workers designed an electrochemical cell capable of performing XAS measurements simultaneously in both transmission and fluorescence modes (Lopez-Astacio *et al.*, 2024 ▸). Bott-Neto *et al.* (2020 ▸) introduced a versatile spectroelectrochemical cell (SEC) capable of supporting a wide range of *in situ* spectroscopic techniques, including FT-IR, Raman, XAS (fluorescence) and ultrafast spectroscopy. Binninger *et al.* (2016 ▸) developed an electrochemical flow cell specifically for *in situ* small-angle X-ray scattering (SAXS) and XAS experiments. Recently, Winzely *et al.* (2024 ▸) demonstrated a cell for *operando* grazing-incidence X-ray absorption spectroscopy (GIXAS) studies. This cell features a dedicated window for fluorescence detection, allowing the detector to be positioned at an optimal angle (*e.g.* 45°) relative to the incident X-ray beam.

Despite recent advancements, several challenges remain in the practical application of electrochemical cells for catalyst studies. First, to capture comprehensively the dynamic structural changes in catalysts, there is a growing need for electro­chemical cells capable of simultaneously collecting both transmission and fluorescence XAS signals, as well as XRD signals. This capability is essential because the structure evolution of materials at the surface and in the bulk can differ significantly. Understanding these differences is crucial for elucidating catalytic mechanisms and optimizing material performance. Secondly, in practical applications, it has been observed that the XAS of some transition metals with absorption edges in the 5–9 keV range can be strongly affected by the water window. This interference can significantly degrade the quality of the data, making it challenging to obtain reliable structural information. Therefore, electrochemical cells should be equipped with additional features to adjust the water window, thereby optimizing the signal-to-noise ratio of the data. Such adjustments can help mitigate the effects of the water window, ensuring that the XAS measurements remain accurate and informative.

To address these challenges, we have designed a specialized *in situ* electrochemical cell which is presented here. This cell is meticulously designed to support *operando* XAS in both fluorescence and transmission modes, as well as *operando* XRD in transmission mode. The main components of the electrochemical cells were made of polyether ether ketone (PEEK) and polytetra­fluoro­ethyl­ene (PTFE), which offer superior stability for electrolytes across a wide pH range 0–14. To demonstrate the broad application potential of this *operando* setup, we showcase its use with a commercial LiCoO_2_ oxide as an OER catalyst. LiCoO_2_ is a well documented OER catalyst in alkaline media (Zeng *et al.*, 2024 ▸; Zeng *et al.*, 2025 ▸). However, recent studies have revealed complexities in its operational mechanism (Xiong *et al.*, 2023 ▸). It has been observed that Li^+^ can leach into the electrolyte solution as Co undergoes oxidation during OER. Conversely, cations present in the electrolyte, *e.g.* Na^+^ or K^+^, can back-intercalate into the bulk of LiCoO_2_ during the reaction, inducing structural phase transitions. This dual phenomenon of chemical valence changes and overall crystallographic transformations makes LiCoO_2_ an ideal candidate for demonstrating the capabilities of our *in situ* experimental setup. By employing *operando* XRD and XAS techniques, we were able to elucidate the dynamic cation intercalation/deintercalation processes into and out of the bulk layered structure of LiCoO_2_ during OER.

## 2. Design of the electrochemical cell

**Figure 1:**
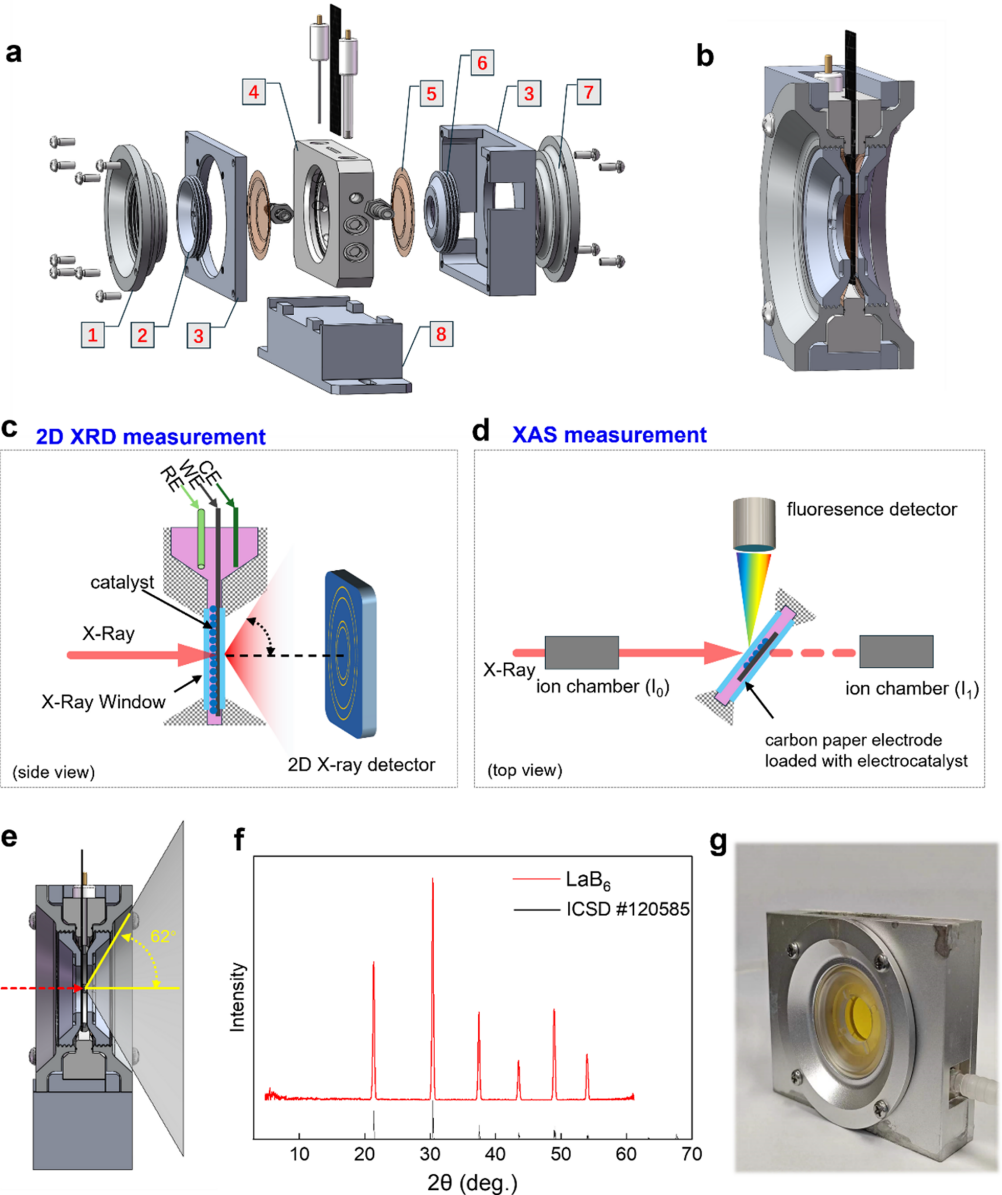
Design of the *in situ* XRD/XAS electrochemical cell. (*a*) Exploded view showing different components of the cell: (1) front rotating cap 1 (large), (2) front rotating cap 2 (small), (3) housing structure, (4) main component of the cell, (5) Kapton membrane, (6) back rotating cap 2 (small), (7) back rotating cap 1 (large) and (8) sample stage. (*b*) 3D rendering showing the intricate design of the cell’s cross-sectional architecture. (*c*) Scheme for 2D XRD measurement. (*d*) Scheme for XAS measurement in both fluorescence and transmission modes. (*e*) Cross-sectional view showing the collection angle of X-ray signals. (*f*) XRD pattern of an LaB_6_ electrode. (*g*) Photograph of the fabricated cell.

Fig. 1 ▸ schematically illustrates the cell design, which features a symmetrical layout. The cell is centered around an 8 cm diameter aperture and incorporates a housing structure to accommodate the various components. It includes a set of larger-diameter outer rotating caps and a set of smaller-diameter inner rotating caps, both with a ring configuration, along with two Kapton membrane windows that facilitate X-ray transmission. The outer caps are affixed to the reaction chamber using a threaded connection and the inner caps are mounted onto the outer caps in the same threaded fashion. A Kapton membrane is held in place between these caps, sealed with multiple O-rings to ensure an airtight seal.

The cell’s design features two 3 mm diameter holes at the top, strategically positioned for the insertion of the counter and reference electrodes. In the center, a precisely cut slit of 10 mm by 20 mm serves as the location for the working electrode (WE). Commonly, the WE is a strip of carbon paper, upon which a square area of 10 mm by 10 mm is coated with the catalyst through a drop-casting method. This configuration ensures efficient electrode placement and optimal catalyst application, thereby facilitating effective electrochemical measurements. The selection of these dimensions, *i.e.* 8 cm diameter aperture, 3 mm diameter holes for the counter and reference electrodes and a 10 mm × 10 mm catalyst-coated area on the working electrode, was based on a combination of practical considerations, compatibility with existing electrodes and alignment with our prior studies (Qi *et al.*, 2023 ▸; Zeng *et al.*, 2024 ▸).

To enhance the applicability of this setup and address the aforementioned challenges, we have implemented the following improvements. In addition, several technical challenges were addressed to ensure the reliability and accuracy of the measurements (Muñoz-Páez *et al.*, 1995 ▸; Bare *et al.*, 2007 ▸; Shimizu *et al.*, 2022 ▸; Fulton *et al.*, 2012 ▸).

### 2.1. Enable XAS measurements in transmission and fluorescence modes

The fluorescence XAS measurement, in particular, requires a near 90° angle between the incident X-ray beam and the fluorescence X-ray detector. To accommodate this requirement, the cell must have a broad X-ray entry window. As shown in Fig. 1 ▸(*d*), by creating a 45° angle slope on the X-ray receiving window, a large (3 mm × 3 mm) area of access to the sample is provided. This design enables the use of both transmission and fluorescence XAS modes simultaneously and provides several benefits:

(i) *Adaptability in measurement mode based on catalyst concentration.* It is widely recognized that the XAFS measurement modes should be tailored according to the elemental concentration of the sample. For instance, transmission mode is recommended for catalysts containing above 5 wt% of the target element, while fluorescence mode should be employed for levels below 5 wt%. Thus, the design of our cell offers versatility.

(ii) *Simultaneous collection of transmission and fluorescence XAS signals.* The cell enables the simultaneous collection of transmission and fluorescence XAS signals using an ion chamber and a fluorescence detector, respectively. Indeed, some XAS beamlines, such as the BL11B beamline at the Shanghai Synchrotron Radiation Facility (SSRF), offer such capability. Transmission mode yields insights into the bulk structure of the catalyst, while fluorescence mode provides more detailed information about the surface structures. The combination is significant for investigating the surface structural evolution and reconstruction of catalysts. This aspect of our research warrants further exploration in future studies.

### 2.2. Broad signal collection angle for 2D XRD measurement

In XRD analysis, structure refinement using Rietveld fitting typically requires as many diffraction peaks as possible to enhance the accuracy of the fitting. Therefore, the electrochemical cell must incorporate a broad angle for signal collection. To address this issue, we engineered the screw cap and pressure ring with a large-area hollow design. When assembled, this design allows for the collection of diffraction rings up to 62°. As shown in Figs. 1 ▸(*e*) and 1 ▸(*f*), this feature ensures a broad angle of signal collection, which is particularly beneficial for capturing higher-angle XRD signals that are critical for a comprehensive structural analysis.

### 2.3. Control the thickness of the water window

Since the *in situ* cell is capable of both XAS and XRD measurements in transmission mode, the X-ray path traverses not only the reactive material on the electrode but also the surrounding aqueous electrolyte, whether in strongly acid or alkaline conditions, before reaching the detector. Water can significantly absorb X-rays, leading to a decrease in their intensity. This absorption effect is particularly pronounced for X-rays with energies in the 5–9 keV range, which is relevant for the XAS of many transition metals. Therefore, in *operando*X-ray measurements, it is crucial to control the thickness of the water electrolyte to minimize this absorption loss.

**Figure 2:**
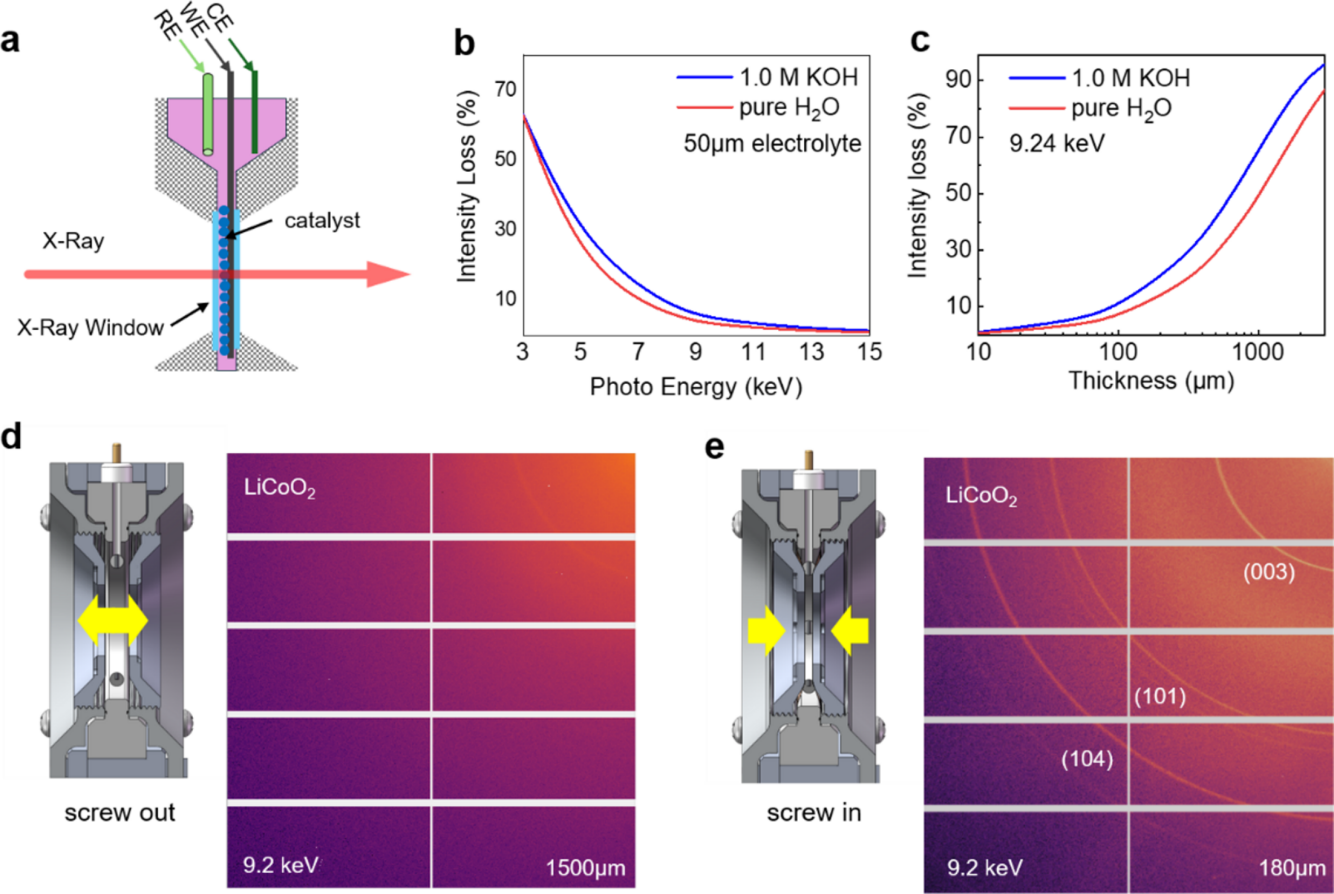
Function for adjusting the water window. (*a*) Schematic illustration of X-ray penetration through the catalyst and electrolyte. (*b*) Intensity loss (%) for X-rays with different energies after penetrating 50 µm thickness of H_2_O or 1.0 *M* KOH electrolyte. (*c*) Intensity loss (%) for X-rays with a characteristic 9.2 keV energy after penetrating different thicknesses of H_2_O or 1.0 *M* KOH electrolyte. (*d*)–(*e*) Schemes for adjusting the thickness of the water window by screwing in (180 µm) and out (1500 µm) the small rotating caps (components 2 and 6) to obtain better diffraction signals. The testing was conducted on a laboratory-based XRD spectrometer using an In–Ga metal-jet X-ray source with a characteristic energy of 9.2 keV.

As illustrated in Fig. 2 ▸, we conducted a detailed analysis to quantify the impact of water absorption on X-ray intensity. We assumed a water electrolyte thickness of 50 µm and calculated the decrease in X-ray intensity for different X-ray energies. The results indicated that for X-rays with energies between 3 and 9 keV, the intensity loss due to water absorption can range from 60% to 10%. Specifically, we demonstrated *operando* XRD using a laboratory-based X-ray diffractometer with a characteristic X-ray energy of 9.2 keV. Our calculations revealed that when the KOH electrolyte thickness exceeds 100 µm, there could be more than 10% X-ray intensity loss due to water absorption. This finding underscores the necessity of designing an *operando* cell that allows for precise adjustment and control of the water window thickness.

To tackle this challenge, we have engineered a screw cap for the electrochemical cell [Figs. 2 ▸(*d*) and 2 ▸(*e*)], which includes an exterior thread that interlocks with the interior thread of a pressure ring. By rotating the caps, the thickness of the water window can be precisely controlled. This method of adjusting the position through threaded structures has been previously reported, notably in a fluorescence XAFS cell design by Paparoni *et al.* (2024 ▸), where the rotation of a threaded glassy carbon electrode was used to regulate the liquid layer’s thickness between the catalyst and the X-ray transparent window. Our design ensures an unobstructed transmission path for X-ray beams, thus facilitating both fluorescence and transmission XAS measurements.

### 2.4. Management of gas bubbles on electrode surface

During many electrocatalytic processes, such as the oxygen or hydrogen evolution reactions, gaseous products like O_2_ or H_2_ form at the catalyst–electrolyte interfaces, which may further adhere on the electrode surface, generating gas bubbles. Based on our experimental experience, especially during OER tests, we have observed that when the applied potential exceeds 1.55 V *versus* reversible hydrogen electrode (RHE), a significant number of O_2_ gas bubbles are generated on the electrode surface due to water oxidation. These bubbles can accumulate and displace the electrolyte, which not only reduces the electrochemical performance of the catalyst but also temporarily disrupts the X-ray intensity. This disruption can affect the reliability of our *in situ* measurements. To address this challenge, the electrochemical cell was designed with an integrated electrolyte flow system, facilitating the removal of gaseous byproducts and maintaining a clear path for X-ray analysis. As shown in Fig. S1, the flowing electrolyte mode is essential for several reasons: it enhances the diffusion kinetics, ensures a continuous supply of fresh electrolyte to the electrode surface and removes the generated O_2_ bubbles to prevent their accumulation. During *operando* XAS or XRD measurements, we take several steps to ensure the system operates smoothly. We conduct regular visual inspections of the flow path and electrode surface to identify any visible signs of O_2_ bubble blockage. We also carefully adjust the flow rate to ensure that the peristaltic pump is functioning correctly and that the flow rate remains consistent throughout the experiment. Moreover, we closely monitor the electrochemical performance of the system. Any sudden changes, such as increased overpotential or decreased current density, can be indicative of issues affecting the electrolyte flow.

### 2.5. Corrosive resistance and good sealing.

Electrocatalytic reactions often take place under conditions that are either highly acidic or highly alkaline, requiring the use of materials capable of enduring these harsh environments and allowing X-ray penetration. For this project, the cell body is crafted from PEEK, a robust polymer that is impervious to strong acids and alkalis and has demonstrated long-term operational stability. However, the choice of Kapton film for a window material that is transparent for X-ray beams, while showing adequate resistance to acidic conditions, is still vulnerable to rapid degradation in hot alkaline solutions, potentially leading to rupture. As a result, ongoing material exploration is essential to identify alternatives that combine superior corrosion resistance with the necessary X-ray transparency for *in situ* analysis. The rotating caps, which do not contact the electrolyte, can be made from aluminium alloy, offering a cost-effective and easily machinable alternative to PEEK.

## 3. Experimental

LiCoO_2_ (also denoted LCO) powder was purchased from Sigma Aldrich and used as received.

For the electrochemistry measurements, carbon paper was used to evaluate the OER activities for LiCoO_2_. The catalyst was loaded onto the working electrode by the conventional drop-casting method. A mixture containing 8 mg of LCO powder, 5 wt% acetyl­ene carbon black and 60 µl of Nafion ionomer (5% weight, Ion Power) was dispersed in 200 µl of tetra­hydro­furan (THF). This catalyst ink was drop-cast onto the carbon paper electrode to form a catalyst area of 1.0 cm × 1.0 cm and dried in air at room temperature (25°C). The carbon paper electrode coated with the drop-cast LCO catalyst served as the working electrode, while an Hg/HgO (1.0 *M* KOH) reference electrode and a Pt wire were used as the reference electrode (RE) and counter electrode (CE), respectively. Prior to each experiment, a reversible hydrogen reference electrode (HydroFlex, from Gaskatel, Germany) was used to calibrate the Hg/HgO (1.0 *M* KOH) reference. Electrochemical potentials were converted to the RHE scale. During cyclic voltammetry (CV), the scan rate was set to 10 mV s^−1^.

*Operando* XRD measurements were performed on a home-built XRD instrument equipped with a liquid-metal-jet anode X-ray source (Excillum Metaljet D2 70 kV 250w) with a characteristic energy of 9.2 keV and a Pilatus3R 1M X-ray detector. The *operando* cell was set between the X-ray source and the detector (Li *et al.*, 2025 ▸). The beam was perpendicular to the working electrode. A three-electrode configuration was employed. KOH solution (15 ml, 0.1 *M*) was added to the electrochemical cell through the cavity. All electrodes were connected to a BioLogic SP-50e potentiostat. The WE was stepwise charged from 1.1 to 1.7 V *versus* RHE. For each potential step (50 mV per step), the electrode was potentio­statically held for 10 min, during which XRD profiles (2 min exposure per spectrum) were collected. The interlayer distance (*d* spacing) of LiCoO_2_ can be calculated using Bragg’s law based on the equation 2*d*sinθ = *n*λ, where *d* is the interplanar spacing, θ is the diffraction angle, *n* is the order of the reflection and λ is the X-ray wavelength. The (003) diffraction peak was used for this calculation.

*Operando* XAFS measurements were conducted on the X-ray Absorption Fine Structure for Catalysis (XAFCA) beamline at the Singapore Synchrotron Light Source (SSLS). An Si(111) double-crystal monochromator was employed for XAFS measurement. The X-ray energy calibration was conducted using a cobalt foil as a standard reference to align the characteristic Co *K* absorption edge. An 80% nitro­gen (N_2_) and 20% argon (Ar) gas mixture was used to fill the ionization chamber. All measurements were performed in transmission mode utilizing the ionization chamber. The preparation of the working electrode and the selection of RE and CE were consistent with the *operando* XRD test. KOH solution (15 ml, 0.1 *M*) was added to the electrolyte cavity. The water window was adjusted to be ∼180 µm in thickness during measurement. XAFS data were collected while the WE was stepwise charged from 1.1 to 1.7 V *versus* RHE. For each potential step, the electrode was galvano­statically held for 10 min. XAFS spectra were collected for each potential step.

## 4. Results and discussion

**Figure 3:**
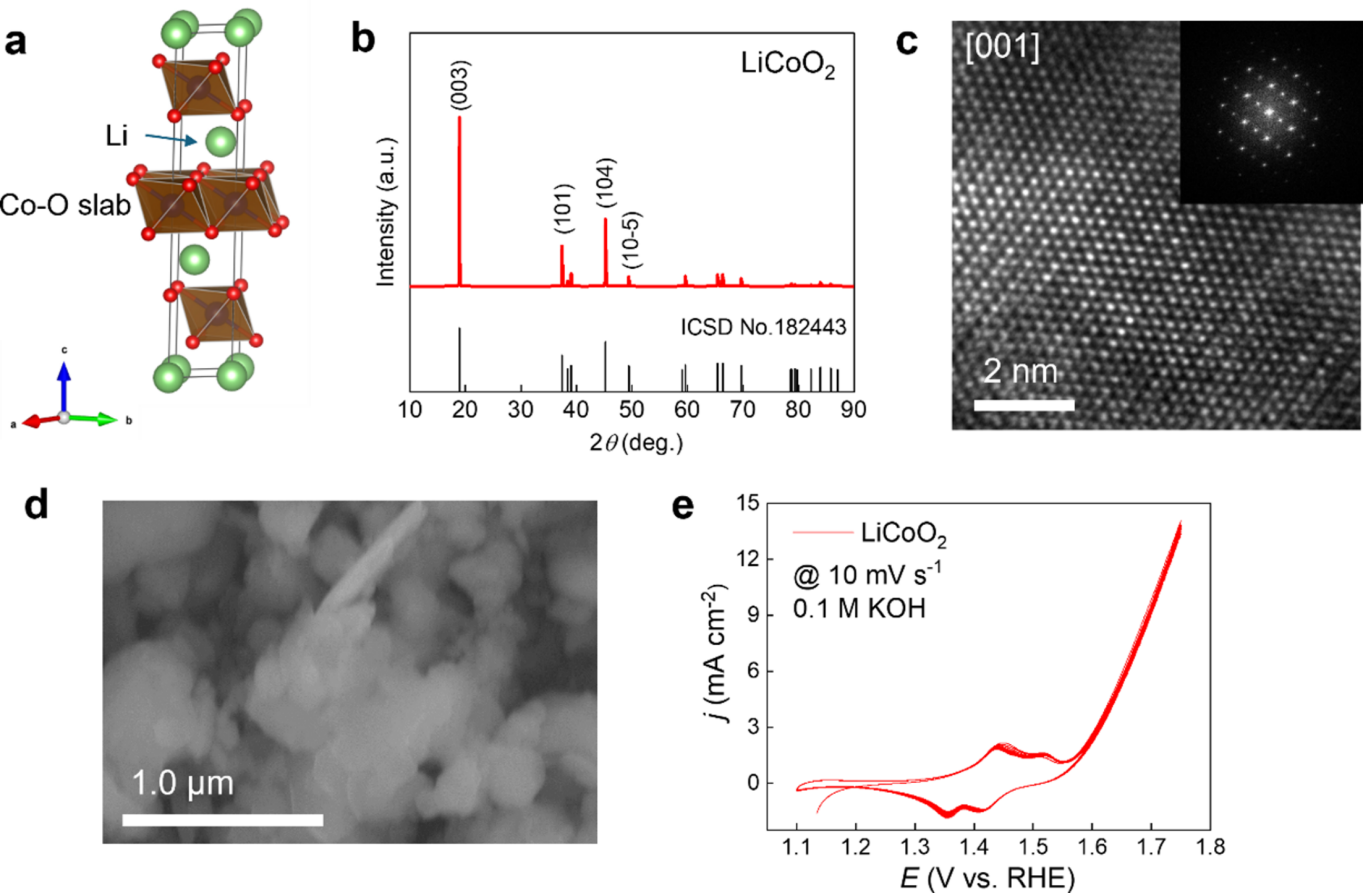
(*a*) Crystal structure of LiCoO_2_. (*b*) XRD pattern, (*c*) TEM image and (*d*) SEM image of commercial LiCoO_2_ oxide. (*e*) CV curves of LiCoO_2_ catalysts in 0.1 *M* KOH at a scan rate of 10 mV s^−1^ for ten cycles.

To validate the utility and reliability of our electrochemical *in situ* cell, we investigated the structural evolution of a layer-structured LiCoO_2_ oxide as OER catalyst in an alkaline medium (Chen *et al.*, 2016 ▸; Zeng *et al.*, 2024 ▸). Recognizing that LiCoO_2_ is a widely commercialized cathode material in Li-ion batteries, there are a considerable number of spent LiCoO_2_ batteries on the market that are earmarked for recycling. The challenge is to reutilize the recycled Li_*x*_CoO_2_ material effectively, since delithiation of LiCoO_2_ results in deficient Li in the layered structure, forming Li_*x*_CoO_2_, which possesses metallic conductivity and has been reported as a promising OER catalyst (Lu *et al.*, 2014 ▸). Therefore, in this work, we selected recycled LiCoO_2_ as the model catalyst and carried out a thorough assessment of its OER catalytic performance. Fig. 3 ▸ presents a series of characterizations, including XRD, TEM and SEM. The LCO exhibited a hexagonal structure with the space group 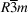. The TEM image, showing the [001] direction, revealed a highly ordered atomic arrangement within the transition metal layers. The LCO particles were uniform in size, with dimensions in the hundreds of nanometres. CV measurements were conducted in a 0.1 *M* KOH solution within the potential range of 1.1 to 1.75 V *versus* RHE. The voltammogram exhibited a pronounced oxidation–reduction peak at approximately 1.4 V *versus* RHE, corresponding to the redox reaction involving the Co^2+/3+^ transition. At a potential of 1.6 V, LiCoO_2_ demonstrates its capability to catalyze water oxidation.

**Figure 4:**
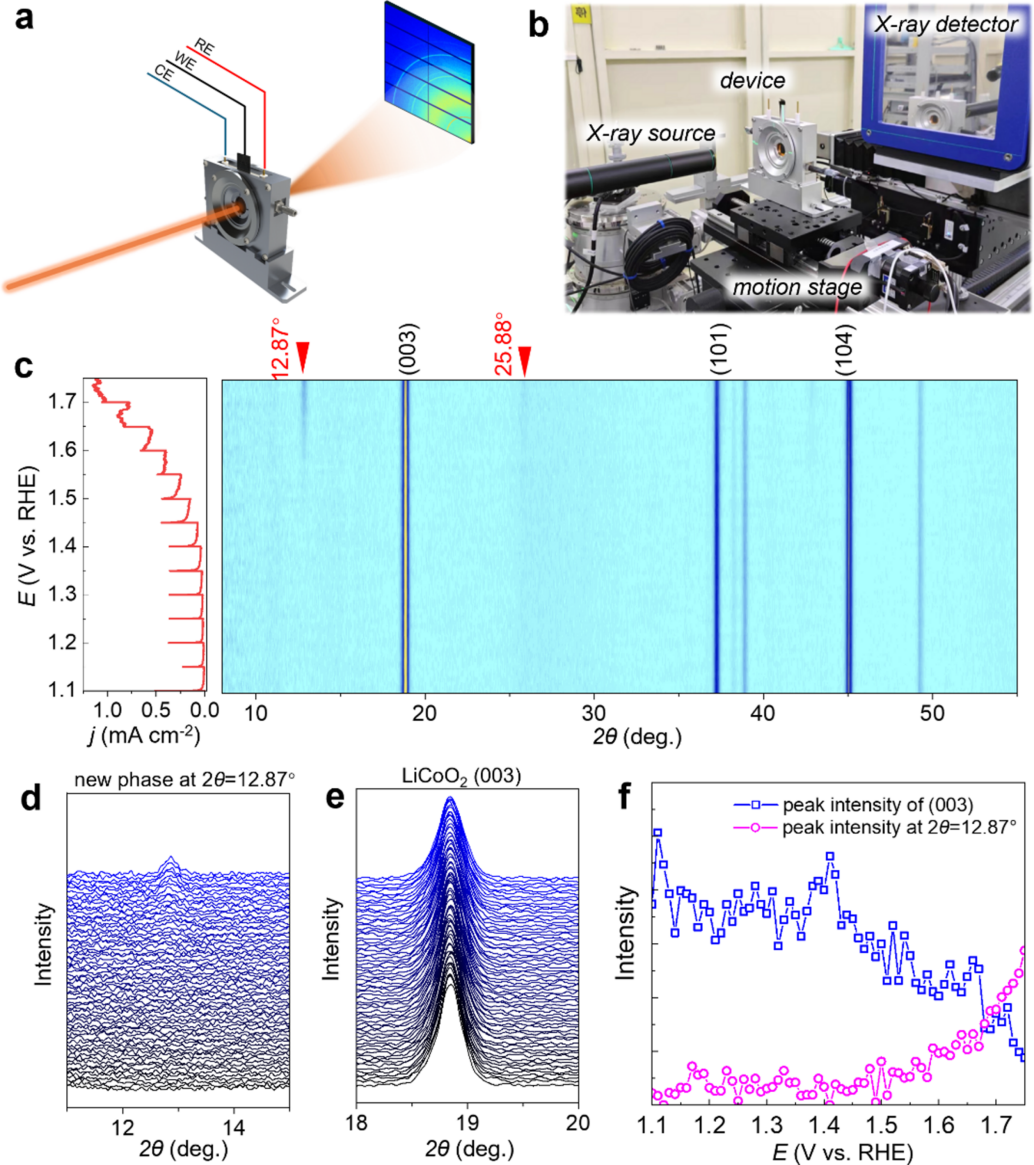
(*a*) Schematic illustration of the working mechanism for *operando* XRD measurements. (*b*) Photograph of the experimental setup. (*c*) Evolution of XRD profiles of LiCoO_2_ by potentiostatic holding at stepwise increased potentials in 0.1 *M* KOH. New peaks emerge at 12.87° and 25.88° above 1.50 V *versus* RHE, as indicated by the red arrows. These peaks can be attributed to a new phase, K_0.3_CoO_2_(H_2_O)_0.4_ (ICSD 154037). (*d*) and (*e*) Enlarged views of (*d*) the new diffraction peak emerging at 2θ = 12.87° and (*e*) the (003) diffraction peak of LiCoO_2_ at 2θ = 18.84°. (*f*) Comparison of peak intensities *versus* electrode potentials for the newly appearing peak (pink) and the (003) diffraction peak of LiCoO_2_ (blue).

To investigate further the structural phase transitions of the LiCoO_2_ catalyst during OER, we employed a laboratory-designed X-ray diffractometer equipped with an In–Ga liquid-metal-jet X-ray source. This setup was coupled with our custom-designed *in situ* cell, enabling real-time examination of the catalyst’s structural evolution under OER conditions. Fig. 4 ▸(*a*) illustrates the principle of our experimental setup, while Fig. 4 ▸(*b*) presents a photograph from the *operando* XRD testing. Fig. 4 ▸(*c*) displays the data analysis from the *in situ* XRD test, which includes both electrochemical and XRD results. We gradually increased the electrode potential from 1.1 to 1.7 V *versus* RHE by 50 mV for each step and held the electrode for 10 min at each step. During the potentiostatic period, time-resolved XRD signals were continuously collected. As shown in Fig. 4 ▸(*c*), the (003) peak of LiCoO_2_, positioned at 18.84°, remained relatively stable in terms of peak position throughout the OER process, whereas the peak intensity decreased gradually. The broadening of the FWHM suggests a reduction in crystalline sizes.

Significantly, the *operando* XRD measurement documented the emergence of new peaks at higher OER potentials. Notably, new peaks appeared around 12.87° and 25.88° above 1.50 V *versus* RHE, both of which could be indexed to a new phase K_0.3_CoO_2_(H_2_O)_0.4_ (ICSD 154037; Inorganic Crystal Structure Database, https://icsd.fiz-karlsruhe.de/index.xhtml). The intensity of these peaks escalated with increasing potential. As depicted in Fig. 4 ▸(*f*), the intensity of the (003) peak begins to diminish around 1.45 V, coinciding with the emergence of the new phase peak at 12.87° around 1.5 V. These findings provide valuable insights into the structural dynamics of LiCoO_2_ during OER, particularly highlighting the formation of a new phase at elevated potentials.

**Figure 5:**
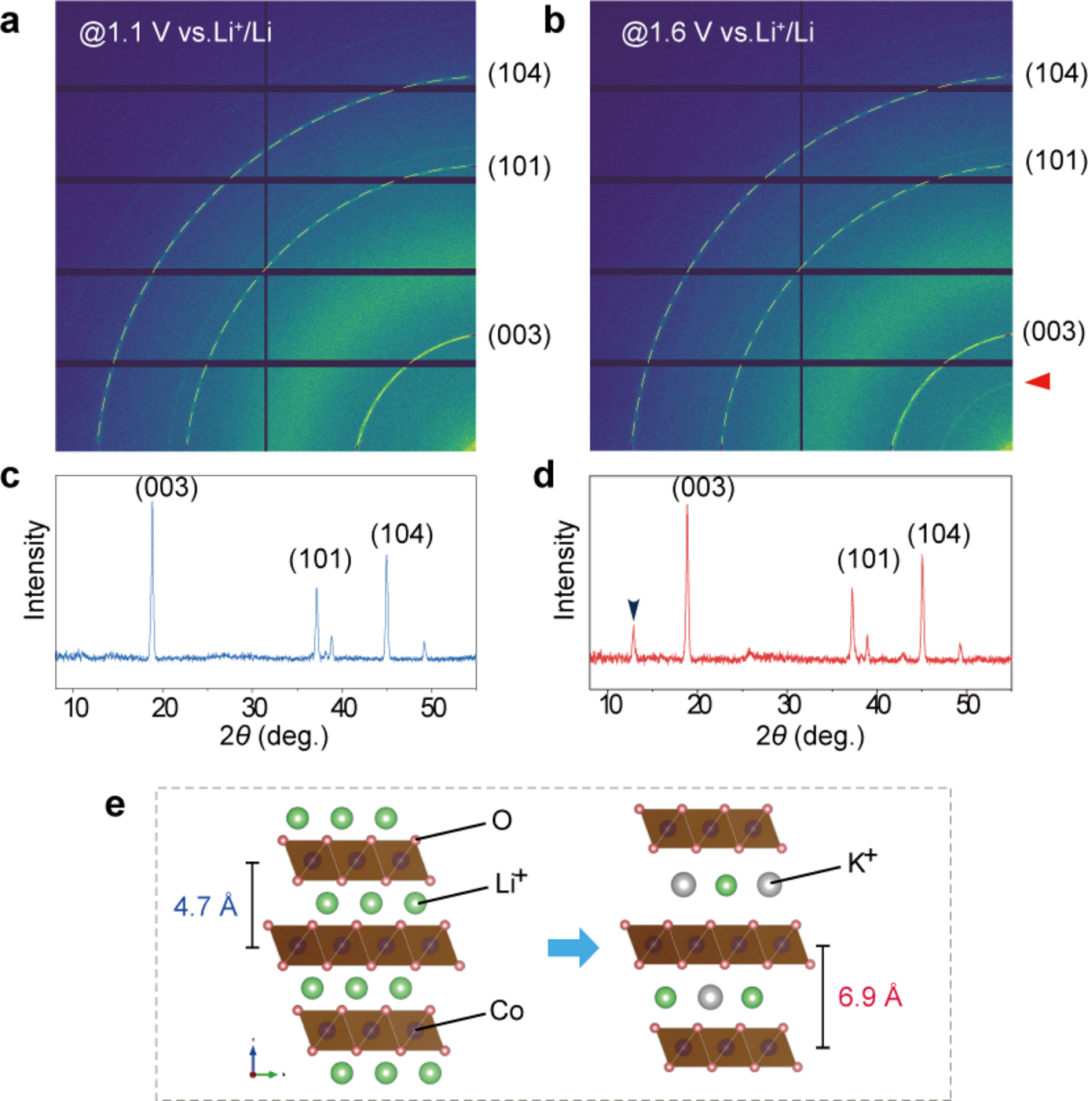
(*a*)–(*b*) Two-dimensional XRD patterns collected by the Pilatus3R 1M X-ray detector (*a*) at OCV and (*b*) at 1.7 V during the *operando* XRD measurement. (*c*)–(*d*) The derived 2θ XRD profiles of LiCoO_2_ (*c*) at OCV and (*d*) at 1.7 V. (*e*) Illustration of the crystal structure changes during OER.

Fig. 5 ▸ compares the raw XRD data obtained at 1.1 and 1.7 V *versus* RHE during the OER process. The pattern obtained at 1.1 V aligns with that of the typical LiCoO_2_ structure (ICSD 182443). In contrast, the pattern obtained at 1.7 V distinctly indicates the formation of a new phase, shown by the emergence of a characteristic peak at 12.87°. Therefore, the *operando* XRD measurement has provided crucial evidence that layered LiCoO_2_ undergoes an unusual cation intercalation phenomenon at high OER potentials. Typically, in battery systems, high potentials correspond to the discharge process, during which Li^+^ ions would deintercalate from the layered structure. There has been no direct evidence to suggest that K^+^ ions from the electrolyte solution, with a larger ionic radius, could intercalate back into the layered structure under such high potentials.

**Figure 6:**
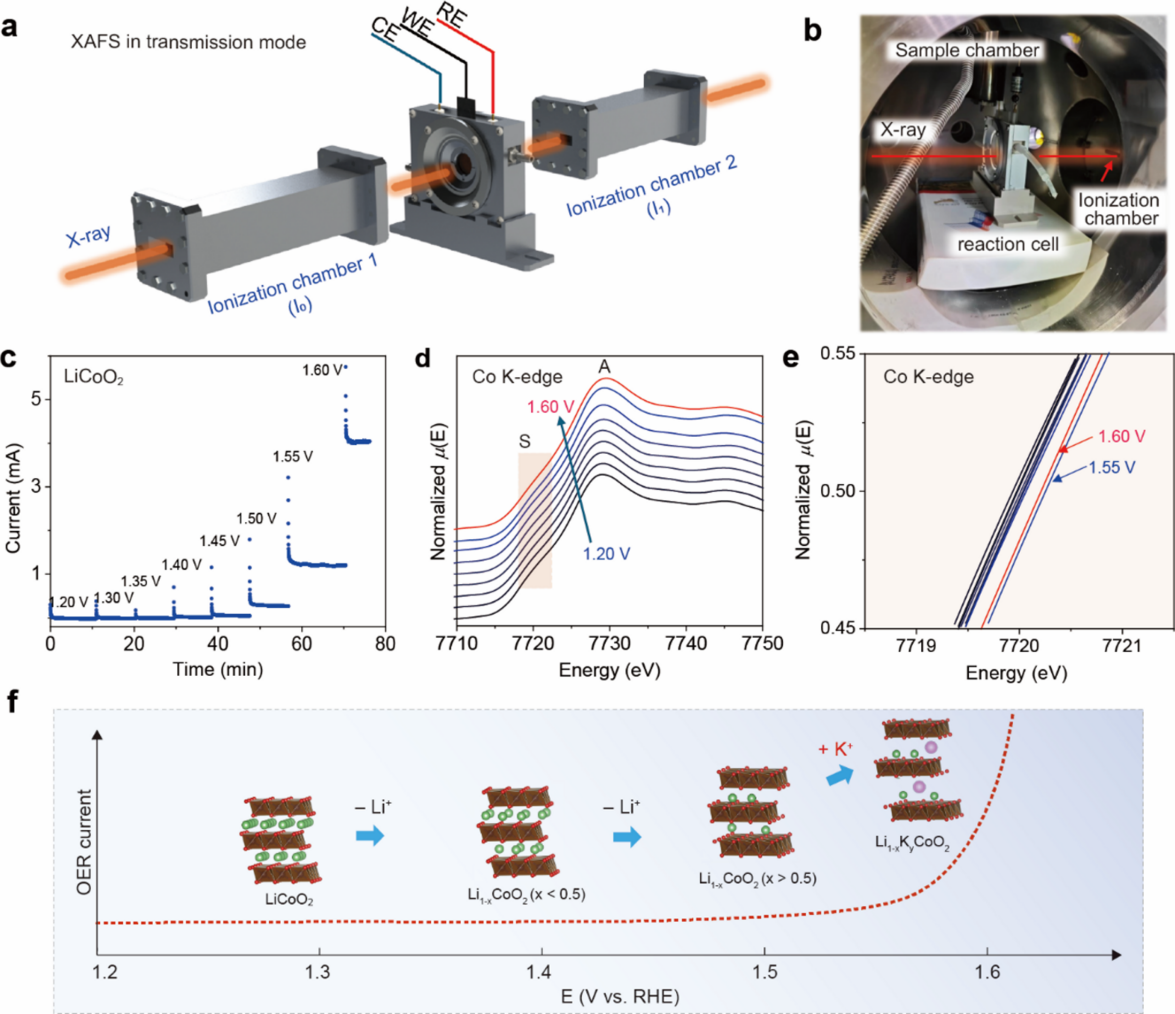
(*a*) Schematic illustration of the *operando* XAFS measurements. (*b*) Photograph of the experimental setup on a synchrotron XAFS beamline. (*c*) Electrochemical profile of the potentiostatic measurements with stepwise increased potentials from 1.2 to 1.6 V *versus* RHE, with each potential step held for 10 min, during which XAFS data are continuously collected. (*d*) *Operando* XANES spectra of LiCoO_2_ at different potentials in 0.1 *M* KOH. (*e*) Shifts of the white line of the Co *K*-edge spectra. (*f*) Schematic illustration of the reaction mechanism depicting the dynamic cation intercalation and deintercalation, and the structural evolution of LiCoO_2_, during OER.

Next, XAFS measurements were conducted in transmission mode, employing an ionization chamber to monitor the X-ray intensity both before and after the operating test electrode. Co *K*-edge XANES spectra were collected for the LiCoO_2_ catalyst. The *operando* testing mechanism and experimental setup are shown in Figs. 6 ▸(*a*) and 6 ▸(*b*), respectively. Fig. 6 ▸(*c*) presents the electrochemical results, which are consistent with those of the*operando* XRD measurements, indicating that the electro­chemical performance is highly reproducible. Fig. 6 ▸(*d*) illustrates the evolution of the XANES spectra with increasing electrode potential from OCV to the OER potential. The XANES features labeled peak A and shoulder peak S are attributed to transitions to unoccupied Co 4*p* states, while the pre-edge at *ca* 7710 V involves transitions from the 1*s* core level to unoccupied *d* states. During the delithiation of LiCoO_2_, the shoulder peak S at *ca* 7715 eV gradually diminishes, as has been reported in the literature (Fakkao *et al.*, 2017 ▸). Upon applying a potential, peak A continuously shifts to higher energies, reflecting the oxidation of Co sites. Meanwhile, the diminishing of shoulder peak S could be associated with the decreased Li^+^ ion concentration within the LiCoO_2_ structure. A distinct shift of 0.74 eV to higher energy is observed at 1.55 V *versus* RHE, suggesting oxidation of the Co sites. Subsequently, a shift of 0.37 eV towards lower energy is detected at 1.6 V *versus* RHE. The first-derivative XANES curves confirm such shifts upon increasing the electrode potential (Figs. S3 and S4). These observations indicate that the valence state of Co initially increases and then slightly decreases during the OER operation.

Combining the evolution trend of the chemical states of Co as shown by the *operando* XANES measurement, together with the abnormal structural changes above 1.5 V *versus* RHE detected by the *operando* XRD measurement, we can deduce a mechanism for the whole reaction process. As illustrated in Fig. 6 ▸(*f*), LiCoO_2_ experiences continuous delithiation during the OER process, accompanied by an increase in the valence state of Co and a corresponding shift of the absorption edge to higher energy. However, when the potential surpasses 1.5 V, K^+^ cations seem to intercalate into the layered structure in a reverse process. This appears to be a spontaneous surface chemical process that results in an expansion of the interlayer spacing from 4.7 Å to 6.9 Å, along with a significant and unexpected decrease in the valence state of cobalt.

Our studies have uncovered simultaneous structural transformations in LiCoO_2_ catalysts during OER under alkaline conditions, which result in an expansion of the layered *d* spacing and the formation of a new phase, K_0.3_CoO_2_(H_2_O)_0.4_. Our findings align with previous reports, highlighting the dynamic nature of LiCoO_2_ during OER. Kim *et al.* (2022 ▸) explored the OER performance of LiCoO_2_ in various alkaline solutions and discovered that the intercalation of larger alkali metal ions (Na^+^, K^+^ and Cs^+^) into delithiated LiCoO_2_ significantly enhances its OER activity. Specifically, the OER activity increased in the order LiOH < NaOH < KOH < CsOH. This enhancement is attributed to the intercalation of these larger cations, which induces structural transformations and alters the electronic properties of the catalyst. Zeng *et al.* (2024 ▸) further employed online mass spectrometry to demonstrate the chemical redox process between high-valence Co sites and water molecules from the electrolyte, which is facilitated by the intercalation of alkali metal cations. For the first time, using our specially designed *operando* cells, we revealed that the intercalation process occurs concurrently with the OER. Specifically, the delithiation of Li^+^ and the intercalation of K^+^ occur successively alongside the OER process. This intercalation drives chemical oxidation and induces structural changes, which are critical for elucidating the underlying mechanisms of OER activity.

## 5. Conclusions

To summarize, this work introduces an innovative *in situ* testing apparatus designed for *operando* XRD and XAFS measurements. The device is engineered to maximize the collection angle of the Debye–Scherrer rings in XRD by optimizing the window angle. A finely tuned screw mechanism is employed to give precise control of the thickness of the water window, ensuring high-quality X-ray transmission for both XRD and XAFS measurements. To address the challenge of gas bubble formation on electrode surfaces that may cause signal fluctuation, an external flow device is integrated into the apparatus, which effectively clears bubbles and significantly boosts the reliability of the *in situ* testing.

To demonstrate the utility of this apparatus, we applied it to the study of LiCoO_2_ as an OER catalyst. Through *operando* XRD and XAFS measurements, the dynamic structural transformation behavior of LiCoO_2_ in an alkaline medium during OER has been elucidated. The experimental results support a dynamic catalytic viewpoint for the OER process at the surface. This application demonstrates the practicality and effectiveness of the apparatus, which we anticipate will greatly contribute to the advancement of research in electrocatalysis.

## Supplementary Material

Additional experimental details and characterizations. DOI: 10.1107/S1600577525005612/art5003sup1.pdf
