# Supplementary material for: Design and application of an electrochemical cell for operando X-ray diffraction and absorption studies for electrocatalysts
Source: J Synchrotron Radiat. 2025 Aug 15;32(Pt 5):1272–81. doi: 10.1107/S1600577525005612 (PMC12416416; doi:10.1107/S1600577525005612)
Supplement: Supplementary file 1 [file s-32-01272-sup1.pdf]

JOURNAL OF SYNCHROTRON RADIATION

Volume 32 (2025)

Supporting information for article:

Design and application of an electrochemical cell for *operando* X-ray diffraction and absorption studies for electrocatalysts

Jiajun Chen, Zhenzhong Li, Huiyan Zeng, Liting Deng, Long Gu, Chao Wang, Chunzhen Yang and Dongbai Sun

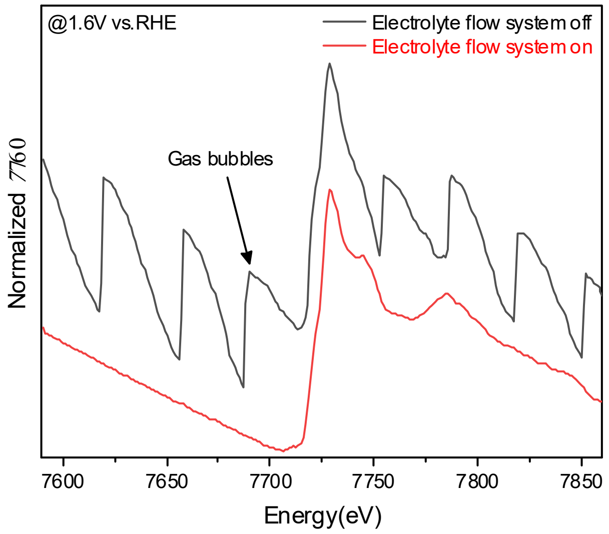

Fig. S1. Comparison of the Co K-edge XAFS measurement when holding the electrode at 1.6 V vs. RHE for oxygen generation with and without turning on the electrolyte flowing system.

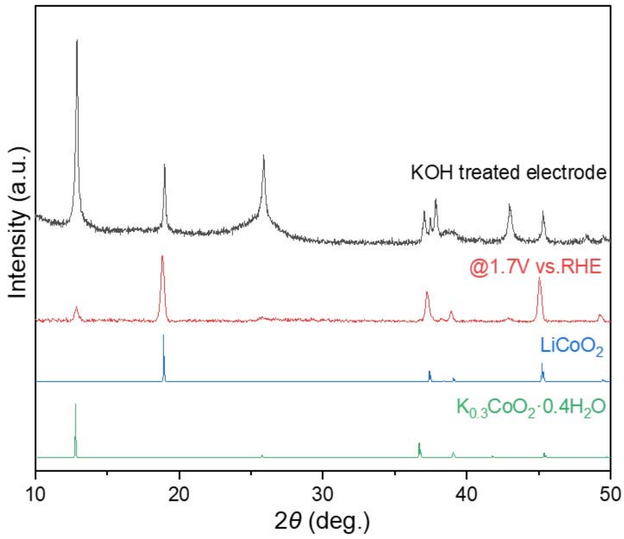

Fig. S2. Comparison of XRD patterns of reacted $LiCoO_2$ electrodes in KOH alkaline electrolyte. The black curve represents the chemically treated sample, which was first electrochemically oxidized and then soaked in a KOH solution for 12 hours. The red curve corresponds to the electrochemically reacted electrode, subjected to potentiostatic measurements in a 0.1 M KOH solution for 2 hours at an applied voltage of 1.7 V vs. RHE. The blue curve depicts the pristine $LiCoO_2$ sample. The green curve serves as a reference for $K_{0.3}CoO_2(H_2O)_{0.4}$ (ICSD # 154037).

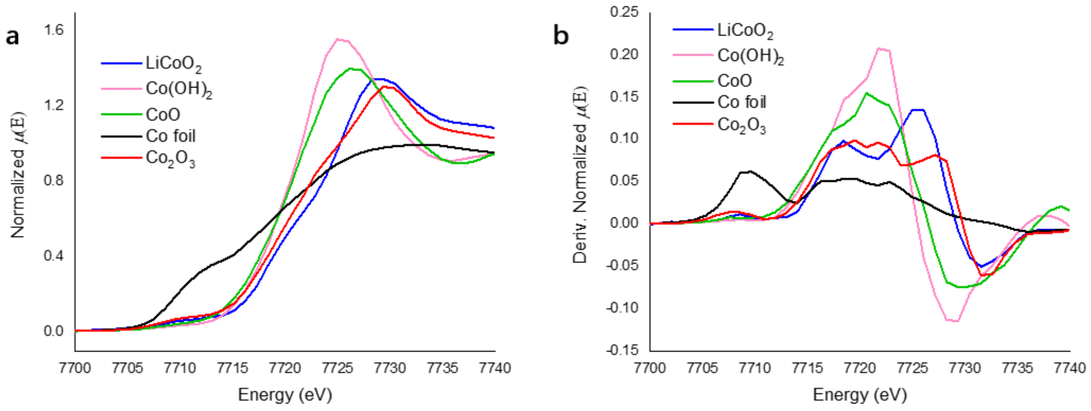

Fig. S3. (a) Co K-edge XANES spectra. CoO, $Co(OH)_2$, Co foil and Co_2O_3 were measured as reference. (b) First derivative of Co K-edge XANES spectra.

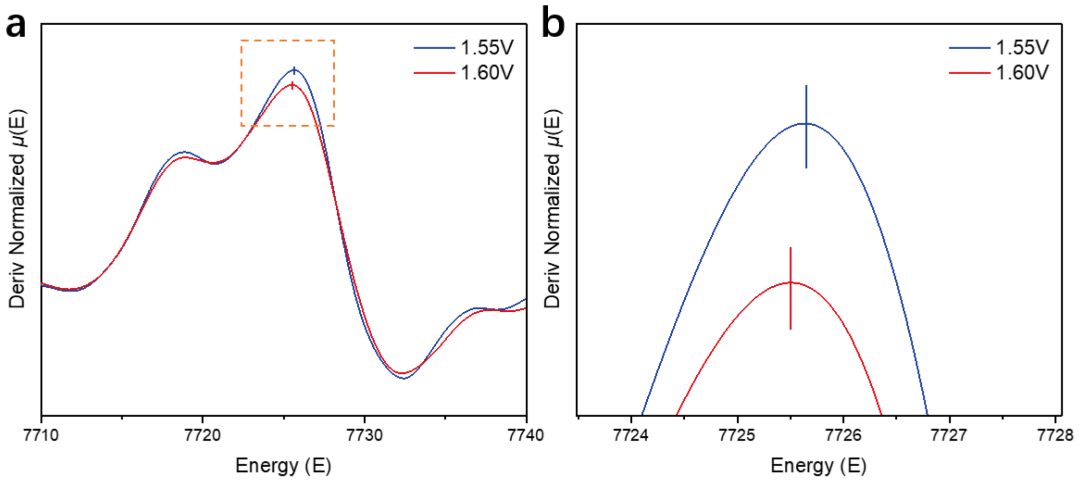

Fig. S4. (a) Comparison of the first derivative Co K-edge XANES spectra measured at 1.55 V and 1.60 V vs. RHE. (b) Zoom-in view of the peak position.

Calculation of the interplanar distance:

The *d*-spacing of the layered structure of $LiCoO_2$ can be calculated by the Bragg's law. The (003) diffraction peak was used for this calculation, based on the following equation:

$$2d \cdot \sin\theta = n \cdot \lambda$$

where d is the interplanar spacing, θ is the diffraction angle, n is the order of reflection, λ is the X-ray wavelength.

For $LiCoO_2$ (003) diffraction peak at 2θ=12.87°:

$$d = \frac{\lambda}{2\sin\theta} = \frac{1.54}{2 \times 0.164} = 4.695\ \text{Å} \approx 4.7\text{Å}$$

For the new phase $K_{0.3}CoO_2 \cdot 0.4(H_2O)$ formed with diffraction peak at 2θ=12.87°:

$$d = \frac{\lambda}{2\sin\theta} = \frac{1.54}{2 \times 0.112} = 6.875\ \text{Å} \approx 6.9\text{Å}$$
